# Cognitive behavioural treatment of insomnia in individuals with persistent persecutory delusions: A pilot trial

**DOI:** 10.1016/j.jbtep.2011.02.004

**Published:** 2011-09

**Authors:** Elissa Myers, Helen Startup, Daniel Freeman

**Affiliations:** a King’s College London, Institute of Psychiatry, UK; b Department of Psychiatry, University of Oxford, Warneford Hospital, Oxford, OX3 7JX, UK

**Keywords:** Persecutory delusions, Paranoia, Schizophrenia, Insomnia, Cognitive behavioural therapy

## Abstract

**Background and Objectives:**

Insomnia is a putative causal factor for persecutory thinking. Recent epidemiological studies show a strong association of insomnia and paranoia. The clinical implication is that reducing insomnia will reduce paranoid delusions. This study, evaluating for the first time the treatment of insomnia in individuals with persecutory delusions, provides a test of this hypothesis. It was predicted that a brief cognitive behavioural intervention for insomnia (CBT-I) for individuals with persistent persecutory delusions and sleep difficulties would not only reduce the insomnia but that it would also reduce the paranoia.

**Methods:**

Fifteen patients with persistent persecutory delusions and insomnia in the context of a psychotic disorder were each individually given a standard-format, four-session CBT-I intervention. Outcome assessments were conducted at pre-treatment, post-treatment and one-month follow-up.

**Results:**

There were no missing data. Following the intervention, significant reductions were found in levels of insomnia and the persecutory delusions. The effect sizes were large, and the changes were maintained at the follow-up. At least two-thirds of participants made substantial improvements in insomnia and approximately half showed substantial reductions in the persecutory delusions. There were also reductions in levels of anomalies of experience, anxiety and depression.

**Limitations:**

The main limitations are the absence of a control group and unblinded assessments. A more methodologically rigorous evaluation of this intervention is now warranted.

**Conclusions:**

These preliminary findings suggest that CBT-I can be used to treat insomnia in individuals with persecutory delusions and that, consistent with the hypothesised causal role, it also lessens the delusions.

## Highlights

► First ever test of the effects of an insomnia reduction intervention for patients with persistent persecutory delusions. ► There were large reductions in both insomnia and persecutory delusions. ► The reductions persisted at one-month follow-up. ► The intervention is brief (4 sessions) and uses basic CBT for insomnia techniques, meaning that it can be easily disseminated. ► The results support the theoretical idea that insomnia is a causal factor in paranoid experiences.

## 1 Introduction

Treatments for psychosis are likely to be improved by targeting single symptoms using theoretically-driven interventions (Foster, Startup, Potts, & Freeman, 2010; Ross et al., in press; Trower et al., 2004). Persecutory delusions are one of the key psychotic experiences, for which improvements in both pharmacological and psychological treatments are needed (see reviews by Garety, Bentall, & Freeman, 2008; Bebbington, Pilling, & Whittington, 2008). In the current report we take a putative causal factor of persecutory delusions identified from recent research and evaluate the effects of an intervention designed to reduce it. Insomnia - difficulties with getting and staying asleep - is common in people with paranoia and is likely to exacerbate psychotic experiences (Freeman, Brugha et al., 2010; Freeman, Pugh, Vorontsova, & Southgate, 2009). Given that there are efficacious cognitive behavioural treatments (CBT) for insomnia these should be evaluated in psychosis populations, and they may well have the significant added benefit of reducing persecutory delusions. This is the aim of the work described in this report.

It is well established that insomnia is a cause of anxiety, depression and anomalies of experience (e.g. Breslau, Roth, Rosenthal, & Andreski, 1996; Morphy, Dunn, Lewis, Boardman, & Croft, 2007; Luby, Frohman, Grisell, Lenzo, & Gottlieb, 1960), which are all factors that raise the risk of persecutory thinking (Freeman, 2007). Four recent studies have evaluated the link between insomnia and paranoia. In the first study it was found in a small group of patients with persecutory delusions in the context of psychosis that 27% had severe clinical insomnia, 27% had clinical insomnia of moderate severity, and 30% sub-threshold insomnia (Freeman et al., 2009). In the same study, there was a strong and significant association of persecutory thinking and insomnia symptoms in a general population group. The second study analysed data from the 2000 British National Psychiatric Morbidity survey, a representative general population sample of over eight thousand people, and found that insomnia was associated with a two to threefold increase in the risk of paranoid thinking (Freeman, Brugha et al., 2010). This association was subsequently closely replicated in an analysis of the 2007 Adult Psychiatric Morbidity Survey of over seven thousand people (Freeman, McManus et al., 2010). Stronger evidence has been obtained from a longitudinal follow-up of 2382 people from the 2000 British National Psychiatric Morbidity survey, in which it was shown that symptoms of insomnia at the first assessment increased both the likelihood of the new development of paranoid thinking and of the persistence of existing paranoid thinking over the next 18 months (Freeman et al., submitted for publication). Insomnia may be a causal factor in both the development and maintenance of paranoid thinking, and it is likely that in some instances a vicious cycle occurs, with paranoid fears, inactivity, and sleep difficulties exacerbating each other. In sum, the link between insomnia and paranoia is theoretically plausible and is supported by an emerging empirical literature. The clear next step is to test the effects of reductions of insomnia on paranoid thinking.

Studies of insomnia show that it is associated with a negative impact on individuals’ quality of life, health, relationships, ability to perform at work and enjoyment of leisure activities (Smith, Huang, & Manber, 2005). Chronic insomnia is often poorly managed and treatment of chronic insomnia with pharmacotherapy remains controversial because of issues of tolerance and dependency (Espie et al., 2006; Morin et al., 1999; NICE 2004). In insomnia linked with schizophrenia, treatment often involves antipsychotics and sedative hypnotics which are only partially effective in the long term, and are often associated with a daytime “hangover” effect, which can contribute to poor global functioning (Kantrowitz, Citrome, & Javitt, 2009). However, a large body of research, outside of psychosis groups, has found that non-pharmacological cognitive behavioural interventions (CBT-I) are highly effective (Espie et al., 2006; Jacobs, Pace-Schott, Stickgold, & Otto, 2004; Morin et al., 1999; Smith et al., 2005).

The present investigation aimed to provide an initial evaluation of CBT-I intervention for people with persistent persecutory delusions and co-morbid chronic insomnia. The main prediction was that the insomnia intervention would lead to a reduction in both insomnia and the delusions. The secondary prediction was that the insomnia intervention would also reduce anxiety, depression and anomalies of experience, which are potential mediators of the relationship between insomnia and paranoia. The study provides the first direct evaluation of the effects of an insomnia intervention for individuals with persecutory delusions and was therefore an uncontrolled pilot study of potential efficacy. We targeted patients with delusions that had not responded to current treatments (i.e. a persistent symptom group) in order to strengthen claims for the effects of the intervention.

## 2 Method

The study hypotheses were tested in a pilot study using an open uncontrolled trial methodology based on Harvey, Sharpley, Ree, Stinson, and Clark (2007). Ethical approval for this study was obtained from an NHS research ethics committee.

### 2.1 Participants

15 patients with persecutory delusions and insomnia participated. Criteria for inclusion in the trial were: primary diagnosis of schizophrenia, psychotic disorder, psychosis, schizoaffective disorder or delusional disorder, as indicated by clinical case notes; current experience of persecutory delusions as defined by Freeman and Garety (2000); persecutory delusions that had persisted, despite treatment, for six months or longer; sleep difficulties lasting for one month or longer; medication stable for at least one month prior to taking part in the study; and aged between 18 and 65. Exclusion criteria were: unable to give informed consent; not wanting help for sleep problems; currently involved in any other cognitive behaviour therapy; and a primary diagnosis of drug or alcohol dependence. Participants were recruited from outpatient services.

### 2.2 Intervention

All participants were offered a four-session individual CBT-I intervention (see Table 1). Each session lasted approximately 1 h. The aim was to deliver them weekly, though for some participants it took eight weeks to complete four sessions. The main techniques, standard for CBT sleep interventions, were taken from four main sources: Espie (2006); Freeman and Freeman (2010); Harvey et al., (2007); Meir and Kryger (2004). Initially the sessions focussed upon psycho-education about sleep difficulties, assessment of the triggering and maintenance of sleep difficulties, and goal-setting. Based upon the assessment, the active therapeutic techniques that were used included sleep hygiene, stimulus control therapy (e.g. setting appropriate and regular sleep times, not doing anything else in the bed or bedroom apart from sleeping, not staying in bed if not able to sleep for longer than 20–30 min, stopping daytime naps), relaxation, and, less often, cognitive techniques addressing unhelpful beliefs and attitudes about sleep, attentional bias, monitoring, and safety behaviours. The intervention was deliberately simplified, with the principal therapeutic technique being stimulus control i.e. learning to associate bed with sleep. Participants were also given written information as part of the intervention in the form of a newly-devised handbook to read between sessions and on completion of the intervention. Therapy was carried out by EM, supervised by DF, but adherence and competence was not formally assessed. The persecutory delusions were not discussed in the sessions.

### 2.3 Addendum to design

A baseline assessment had not been included in the original trial design in order not to deter potential participants from taking part in this initial evaluation. However after eight participants had been through the intervention indicating positive outcomes, we enhanced the design for the remaining seven patients with the addition of a baseline measure one or two weeks prior to the pre-treatment measure. The baseline measure included the same set of outcome measures outlined below, so that the stability of the participants’ symptoms could be assessed. Ethical approval was obtained for this addendum to the design.

### 2.4 Outcome measures

#### 2.4.1 Insomnia Severity Index (ISI; Bastien, Vallieres, & Morin, 2001)

The ISI includes seven self-rated items evaluating the perceived severity of insomnia on a five-point scale ranging from 0 (*not at all*) to 4 (*very much*), according to the perceived degree of severity. The total score is obtained by summing the seven items, for a possible total score ranging from 0 to 28. A higher score indicates a greater insomnia severity. The ISI indicates sleep difficulties involving both day and night functioning and covers DSM-IV insomnia criteria (American Psychiatric Association, 1994). Scoring guidelines are as follows: 0–7, absence of clinically significant insomnia; 8–14, sub-threshold insomnia; 15–21, moderate clinical insomnia; and 22–28, severe clinical insomnia. The ISI was found to have adequate internal consistency and is a reliable self-report measure to evaluate perceived sleep difficulties (Bastien et al., 2001).

#### 2.4.2 Pittsburgh Sleep Quality Index (PSQI; Buysse, Reynolds, Monk, Berman, & Kupferet, 1988)

The PSQI is a self-rated questionnaire which assesses sleep quality and disturbances (Buysse et al., 1988). Nineteen individual items generate seven “component” scores: subjective sleep quality, sleep latency, sleep duration, habitual sleep efficiency, sleep disturbances, use of sleeping medication, and daytime dysfunction. Each item is rated on a scale from 0 (no difficulty) to 3 (severe difficulty). The seven component scores are combined to produce a global score (range of 0–21). A PSQI global score >5 is considered to be suggestive of significant sleep disturbance. Psychometric properties of the PSQI were assessed over an l8-month period with “good” sleepers (healthy subjects, *n* = 52) and “poor” sleepers (depressed patients, *n* = 54; sleep-disorder patients, *n* = 62) (Buysse et al., 1988). The scale has been found to a high degree of internal consistency between the seven component scores and good test-retest reliability (Buysse et al., 1988). Regarding validity, the PSQI was shown to provide a standardised, quantitative measure of sleep quality that quickly identifies good and poor sleepers, and compares favourably with the “gold standard” of clinical and laboratory diagnosis (Buysse et al., 1988).

#### 2.4.3 Green et al Paranoid Thought Scales (G-PTS; Green et al., 2008)

The ‘Paranoid Thought Scales’ assess ideas of persecution and reference over the past month. The measure includes two 16-item scales, one assessing ideas of reference (Part A) (e.g. ‘I spent time thinking about friends gossiping about me’ ‘People have been checking up on me.’) and the other ideas of persecution (Part B) (e.g. ‘Certain individuals have had it in for me.’ ‘I was convinced there was a conspiracy about me’). Each item is rated on a five-point scale from 1 (*not at all*) to 5 (*totally*). Scores on each subscale can range from 16 to 80. Higher scores represent a greater degree of ideas of reference and persecution. Good internal consistency and validity has been established for both scales and their dimensions (Green et al., 2008).

#### 2.4.4 Psychotic Symptoms Rating Scale: Delusions Subscale (PSYRATS; Haddock, McCarron, Tarrier, & Faragher, 1999)

The PSYRATS delusions sub scale is a six-item scale which assesses the preoccupation, distress, duration, conviction, intensity of distress and disruption associated with the delusion. Each item is rated on a five-point ordinal scale from 0–4. The scale is used by a clinician in the format of a structured interview and is designed to rate the participant’s experiences over the last week. An exception to this is item three (conviction) which is rated according to the participant’s belief at the time of interview. The PSYRATS has found to have sound psychometric properties (Drake, Haddock, Tarrier, Bentall, & Lewis, 2007; Haddock et al., 1999).

#### 2.4.5 Cardiff Anomalous Perception scale (CAPS; Bell, Halligan, & Ellis, 2006)

The CAPS consists of 32 self-report items designed to assess perceptual anomalies such as changes in levels of sensory intensity, distortion of the external world, sensory flooding and hallucinations. Participants were asked to rate each item using a no (0)/yes (1) format. A higher score indicates a higher number of perceptual anomalies, scores range from 0 (low) to 32 (high). The internal reliability of the CAPS is good, with a Cronbach’s alpha coefficient of .87 (Bell et al., 2006). Test-retest reliability has also been found to be acceptable (Bell et al., 2006).

#### 2.4.6 Depression Anxiety Stress Scales (DASS; Lovibond & Lovibond, 1995)

The DASS is a set of three self-report sub-scales designed to measure the negative emotional states of depression, anxiety and stress over the past week. For this study only the depression and anxiety sub-scales were used. Each item is rated on a four-point ordinal scale from 0–3. Scores range from 0 (low) to 42 (high) on each 14 item subscale. In a clinical sample of 437 the internal consistency (i.e. Cronbach’s alpha) of both the anxiety and depression scale was high, .96 for the depression scale and .89 for the anxiety scale (Brown, Chorpita, Korotitsch, & Barlow, 1997). Temporal stability was also demonstrated in non-significant test-retest scores over a two-week time period (Brown et al., 1997).

### 2.5 Statistical analysis

All analyses were conducted using SPSS Version 15.0 (SPSS, 2007). Changes in each of the outcome measures across time (pre-treatment, post-treatment, one-month follow-up) were assessed using repeated measures analysis of variance (ANOVA). Where the repeated analysis indicated significant changes, differences between pre-treatment, post-treatment and one-month follow-up were then investigated using pairwise comparisons. Treatment effect sizes were calculated using Cohen’s d statistic (Cohen, 1988): $\text{d}={\text{M}}_{\text{initial}}-{\text{M}}_{\text{post}}/{\text{SD}}_{\text{pooled}}$. Reliable and clinically-significant change was reported for the measures used to test the primary hypotheses concerning reductions in insomnia and delusions. Clinically significant change was defined, using stringent criteria, as pre-to post-intervention change of at least two standard deviations from the original mean (Jacobsen & Truax, 1991), but we also examined symptom reduction by 25% and 50%. Reliable change was measured using the Reliable Change Index (RCI) which was calculated using the formula: $\text{RCI}=\left(\text{X}2-\text{X}1\right)/\text{Sdiff},\text{Sdiff}=\sqrt{2}\times {\text{S}}_{\text{E}}\left(\text{squared}\right),{\text{S}}_{\text{E}}=\text{SDpre}\times \sqrt{1-\text{r}}$ (Jacobsen & Truax, 1991). For the design addendum it was hypothesised that there would not be a significant difference between baseline and pre-treatment on the outcome measures. Statistical analysis to test this hypothesis for this addendum involved administering paired *t*-tests to compare baseline with pre-treatment scores. A power calculation was carried out prior to the start of the trial. The aim was to have at least 80% power to detect an effect size of .96 for a reduction in insomnia (see the meta-analysis of Smith et al., 2005) using a single group *t*-test with a .05 two-sided significance level (Howell, 2007). This would need a sample size of 11 participants. We aimed to recruit 15 patients, to allow for loss of data at follow-up.

## 3 Results

**Fig. 1:**
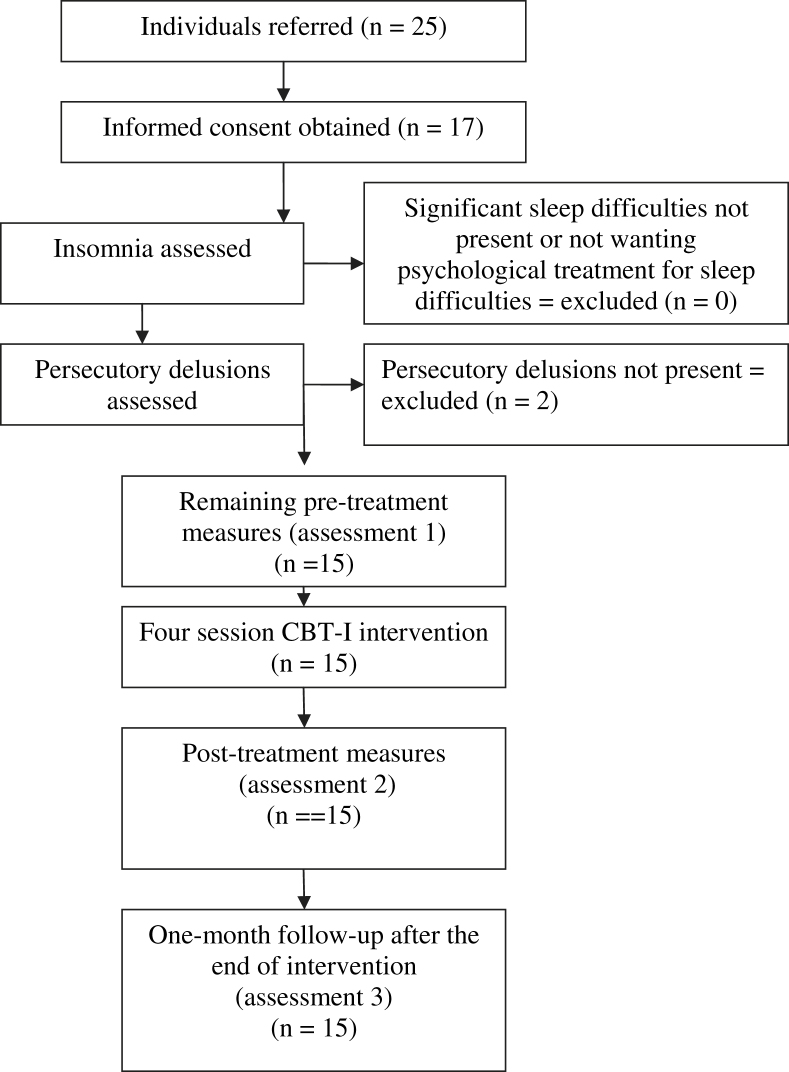
Flowchart of participants progress though the phases of the trial.

There were no missing data in the current investigation. All 15 participants completed the pre-treatment, post-treatment and follow-up assessments (see Fig. 1 for flowchart of participants through the trial). Following an addendum to the study design, seven participants completed an additional baseline measure.

### 3.1 Demographic and clinical characteristics of participants

The demographic details of the participants are summarised in Table 2. It can be seen that this was a relatively older sample, consistent with the criterion of persistent delusions. Typical of this population, most were single and living alone, however there were more women than would normally be expected. Participants who took part in the trial had a case note diagnosis of schizophrenia (*n* = 10), ‘psychotic disorder’ (*n* = 2), delusional disorder (*n* = 1), and ‘psychosis’ (*n* = 1). Symptom scores can be seen in Table 2. All participants in the study fell within the moderate or severe clinical insomnia range on the ISI and in the significant sleep disturbance range on the PSQI. Scores on the G-PTS and PSYRATS indicated that the group had high levels of conviction in the persecutory belief, and delusions that were distressing and preoccupying.

### 3.2 Overview of results

The mean scores of the outcome measures at each time point are displayed in Table 3. It can be seen that there were significant and large reductions over time on all the measures.

### 3.3 Changes in insomnia

#### 3.3.1 Insomnia Severity Index (ISI)

Pairwise comparisons indicated that pre-treatment ISI was significantly different to post-treatment ISI with a mean decrease of 11.8 (*p* < .001; *d* = 2.64) and follow-up ISI with a mean decrease of 10.73 (*p* < .001, *d* = 2.63). Cohen’s d shows that the effect sizes are large. Posttreatment ISI was not significantly different to follow-up ISI (*p* = .20) (see Table 4). Calculation of clinical and reliable change revealed that at post-treatment, 14 participants showed reliable change and 10 made clinically-significant change. At post-treatment, nine participants had reduced ISI scores by 50%, and 14 had reduced scores by 25%.

#### 3.3.2 Pittsburgh Sleep Quality Index (PSQI)

Pairwise comparisons indicated that pre-treatment PSQI was significantly different to post-treatment PSQI with a mean decrease of 8.67 (*p* < .001, *d* = 2.31) and follow-up PSQI with a mean decrease of 8.33 (*p* < .001, *d* = 2.39). Cohen’s d shows that the effect sizes are large. Post-treatment PSQI was not significantly different to follow-up PSQI (*p* = .55) (see Table 4). Calculation of clinical and reliable change revealed that at post-treatment, 14 participants showed reliable change and 12 made clinically-significant change. At post-treatment, nine participants had reduced PSQI scores by 50%, and 13 had reduced scores by 25%.

### 3.4 Changes in persecutory delusions

#### 3.4.1 Green Paranoid Thought Scales: Part A: ideas of reference (G-PTS: A; Green et al., 2008; range 16–80)

Pairwise comparisons indicated that pre-treatment G-PTS: A was significantly different to post-treatment G-PTS: A with a mean decrease of 11.73 (*p* = .001, *d* = .77) and follow-up G-PTS: A with a mean decease of 12.00 (*p* = .003, *d* = .83). Cohen’s d shows that the effect sizes are large. Post-treatment G-PTS: A was not significantly different to follow-up G-PTS: A (*p* = .92) (see Table 5). Calculation of clinical and reliable change revealed that at post-treatment, eight participants showed reliable change and none made clinically-significant change. At post-treatment, two participants had reduced G-PTS: A scores by 50%, and seven had reduced scores by 25%.

#### 3.4.2 Green et al Paranoid Thought Scales: Part B: ideas of persecution (G-PTS: B)

Pairwise comparisons indicated that pre-treatment G-PTS: B was significantly different to post-treatment G-PTS: B with a mean decrease of 18.67 (*p* = .001, *d* = 1.07) and follow-up G-PTS: B with a mean decrease of 19.33 (*p* = .001, *d* = 1.04). Cohen’s d shows that the effect sizes are large. Post G-PTS: B was not significantly different to follow-up G-PTS: B (*p* = .71) (see Table 5). Calculation of clinical and reliable change revealed that at post-treatment, seven participants showed reliable change and five made clinically-significant change. At post-treatment, five participants had reduced G-PTS: B scores by 50%, and eight had reduced scores by 25%.

#### 3.4.3 Psychotic symptoms rating scale: delusions subscale (PSYRATS)

Pairwise comparisons indicated that pre-treatment PSYRATS was significantly different to post-treatment PSYRATS with a mean decrease of 3.27 (*p* < .001, *d* = 1.13) and follow-up PSYRATS with a mean decrease of 4.33 (*p* < .001, *d* = 1.24). Cohen’s d shows that the effect sizes are large. Post-treatment PSYRATS was not significantly different to follow-up PSYRATS (*p* = .25) (see Table 5). Calculation of clinical and reliable change revealed that at post-treatment, 10 participants showed reliable change and two made clinically-significant change. At post-treatment, no participants had reduced PSRATS scores by 50%, and four had reduced scores by 25%.

Though significantly underpowered to examine this issue, changes in persecutory thinking were associated with changes in sleep. GPTS-Part A change (Pre-therapy – Post-therapy) was correlated with ISI change (Pre-therapy – Post-therapy), *r* = .71, *p* = .003, and PSQI change (Pre-therapy – Post-therapy), *r* = .46, *p* = .085. Similarly, GPTS-Part B change was associated with ISI change, *r* = .69, *p* = .005, and PSQI change, *r* = .41, *p* = .129.

### 3.5 Changes in anomalous experience, anxiety and depression

#### 3.5.1 Cardiff Anomalous Perception scale (CAPS)

Pairwise comparisons indicated that pre-treatment CAPS was significantly different to post-treatment CAPS with a mean difference of 4.87 (*p* = .019, *d* = .48) and follow-up CAPS with a mean difference of 4.47 (*p* = .03, *d* = .45). Post-treatment CAPS was not significantly different to follow-up CAPS (see Table 6). Cohen’s d shows that the effect sizes are medium.

#### 3.5.2 Depression anxiety stress scales: anxiety sub-scale (DASS: A)

Pairwise comparisons indicated that pre-treatment DASS: A was significantly different to post-treatment DASS: A with a mean decrease of 10.07 (*p* = .001, *d* = 1.08) and follow-up DASS: A with a mean decrease of 8.33 (*p* = .001, *d* = .85). Post-treatment DASS: A was not significantly increase to follow-up DASS: A (see Table 6). Cohen’s d shows that the effect sizes are large.

#### 3.5.3 Depression anxiety stress scales: depression sub-scale (DASS: D)

Pairwise comparisons indicated that pre-treatment DASS:D was significantly different to post-treatment DASS: D with a mean decrease of 10.27 (*p* = .006, *d* = .69) and follow-up DASS: D with a mean decrease of 7.07 (*p* = .05, *d* = .45). Post-treatment DASS: D was not significantly different to follow-up DASS:D (*p* = .18) (see Table 6). Cohen’s d shows that the effect sizes are medium.

### 3.6 Addendum to design

Baseline data were available for seven participants. It can be seen in Table 7 that there were no changes in the outcome measures prior to the intervention.

### 3.7 Medication

Fourteen participants were taking neuroleptic medication and one participant was not. All those taking medication were on atypical antipsychotics. Converting the antipsychotic doses to chlorpromazine equivalents, six were on low doses of medication (200 mg chlorpromazine or less), seven were on medium doses (201–400 mg chlorpromazine), and one was on a high dose (>400 mg chlorpromazine). Seven participants were also taking anti-depressant medications, and one of these individuals was also prescribed an anxiolytic. Of those taking medication, 13 participants had no change during the period of the trial, consistent with the short length of duration of the trial and the already lengthy treatment history. One participant had a change from 300 mg of Quetiapine to 350 mg of Quetiapine between the post-treatment and follow-up assessment.

### 3.8 Adverse events

No participant had to be withdrawn from the trial, and there were no adverse events. One participant who received CBT-I was admitted as an inpatient to a psychiatric hospital following suicidal thoughts, but the participant and care team did not believe that this was linked to the insomnia intervention and the suicidal ideation had been present prior to entering the trial.

## 4 Discussion

We have reported the first test of an insomnia intervention for individuals with persistent persecutory delusions. The results are very promising. Across all the different outcome measures the results were consistent. There were substantial and significant reductions in insomnia and persecutory delusions. The sleep intervention, which did not discuss the delusions, nevertheless decreased paranoia, which is consistent with a causal role for insomnia in the maintenance of psychotic symptoms. This was a group of patients with long-term and significant difficulties, who would be considered as difficult to treat, but who took very well to a targeted intervention that focussed on a key concern. We recommend more systematic assessment and treatment of sleep difficulties in patients with persecutory delusions. However there is a clear caveat. This was a pilot study, with no control group and unblinded assessments. A randomised controlled trial is now required.

All the participants completed the four session intervention. The results clearly indicate that following the CBT-I intervention, the participants reported significant reductions in insomnia as measured by both the Insomnia Severity Index (ISI) and the Pittsburgh Sleep Quality Index (PSQI). The effect size of the reductions was large. More than two-thirds of participants showed both reliable and clinically-significant change following the intervention. Around half of participants made a 50% improvement and the majority of participants made a 25% improvement on the measures of insomnia. The effect sizes found in the current study are comparable with those found in more rigorous clinical trials of non-pharmacological treatments for insomnia (Morin et al., 1999), and those found in other trials of CBT-I for co-morbid insomnia (Smith et al., 2005). The benefits of the intervention were maintained at the one-month follow-up, which suggests at least a short-term durability of the treatment on insomnia, which is again in keeping with previous treatment studies of non-pharmacological treatments of insomnia (Morin et al., 1999).

Despite the persecutory delusions not being a topic of discussion in the intervention sessions, an improvement in sleep resulted in a lessening of paranoid experiences. The results revealed that following the CBT-I intervention there was a significant reduction in paranoia as measured by the Green et al Paranoid Thoughts Scale (G-PTS) Part A (ideas of reference), G-PTS Part B (ideas of persecution) and the Psychotic Symptoms Rating Scale (PSYRATS) at post-treatment and one-month follow-up. The effect sizes were large. Approximately one half of the participants showed substantial reductions in paranoia on the basis of reliable and clinically-significant change. A small number of participants improved by 50% and almost half of the participants made a 25% improvement on measures of paranoid thinking. These findings are consistent with a recent trial of the treatment of insomnia with sodium salt or y-hydroxybutyric acid (SXB) in eight patients with insomnia linked to schizophrenia (Kantrowitz et al., 2009).

Our introduction of a small baseline period into the study supported the idea that the participant group had persistent symptoms and were not on a path of natural recovery, though this can only be truly tested with a trial design that includes a control group.

It is also of note that the CBT-I intervention was also associated with reductions in levels of anomalies of experience, anxiety and depression. The effect sizes for these reductions were moderate to large, and are similar to those from previous research into CBT-I treatment for insomnia co-morbid with other psychiatric conditions (e.g. Smith et al., 2005). Our interest in these variables was due to their being plausible routes that insomnia may contribute to the occurrence of paranoid thinking. In previous cross-sectional research there were indications that affective disturbance mediates the relationship between insomnia and paranoia (Freeman et al., 2009; Freeman, Brugha et al., 2010). In essence, reductions in anxiety, depression, and anomalies of experience are likely to lead to reductions in persecutory delusions (Freeman, Freeman, & Garety, 2008; Foster et al., 2010).

We have highlighted basic elements of the design of this study that limit the strength of conclusions that can be made at this stage of development of the intervention. We also note that there was only a single therapist, who also carried out the assessments, though these measures were self-report as well as interviewer rated. There was no assessment of therapist adherence or competence. However the simplified intervention we have devised will have the advantage of being easily administered by other therapists. The intervention does not rely on complex formulations or difficult therapeutic manoeuvres. A further weakness is the multiple statistical testing with a relatively small sample size, though confidence is gained by all the measures showing similar changes. Given that there is a substantial evidence base for cognitive behavioural insomnia interventions, we recommend their use for patients with psychosis reporting such difficulties. Our results do not indicate that their effects will be any different for patients with psychosis. Indeed, they hold the intriguing promise of improving psychotic symptoms such as persecutory delusions.

## Conflict of interest

None

## Role of funding source

Funding sources had no input into the design or report of the trial.

## Figures and Tables

**Table 1:** Description of the CBT intervention.

Session Number	Components of Session	Details of Session
Session 1	Psycho-education	What are sleep problems?
		How common are sleep problems?
		What are the effects of sleep problems?
		How much sleep do I need?
		What causes/maintains sleep problems?
	Formulation	What is stopping me sleep?
		Formulation examining thoughts, emotions, physiological symptoms and behaviours associated with insomnia.
	Goal Setting	Goal-setting
		Motivation to change
	Sleep Diary	Sleep diary rationale and practice
Session 2	Sleep Hygiene	Tackling sleep problems
		Lifestyle factors that affect sleep
		Bedroom conditions
		Changes in lifestyle I can make and sleep preparation
	Stimulus Control	Associating bed with sleep
		Bedtime routine
		Wind-down routine
Session 3		Relaxation
		Overcoming sleep–related worry
		Techniques to combat sleep–related worries
		Activity during the day
		Giving up trying to sleep (paradoxical intention)
Session 4	Sleep Review	What have I learnt?
		Revisit goals
		Which techniques worked for you?
		Which techniques didn’t work for you?
	Relapse Prevention	What strategies can I use to help improve my sleep in the future?

**Table 2:** Demographic data.

		All Participants
		(*N* = 15)
Gender		
	Female	9 (60%)
	Male	6 (40%)
Mean age in years (SD)		45.47 (11.28)
	Minimum	20
	Maximum	64
Ethnicity		
	White	7 (46.7%)
	Black Caribbean	1 (6.7%)
	Black African	2 (13.3%)
	Black Other	3 (20%)
	Other	2 (13.3%)
Education		
	GCSE	8 (53.3%)
	AS/A-Level	2 (13.3%)
	Diploma/foundation degree	1 (6.7%)
	Degree	3 (20%)
	Post-graduate Diploma	1 (6.7%)
Marital Status		
	Single	11 (73.3%)
	Divorced/separated	4 (26.7%)
Cohabiting		
	No	15 (100%)
	Yes	0 (0%)

**Table 3:** Means and standard deviations (in parentheses) for main outcome measures.

	Pre–treatment mean score (SD)	Post–treatment mean score (SD)	One-month follow-up mean score(SD)	F	*p*-value
Primary measures					
Insomnia Severity Index	20.93	9.13	10.20	53.87	<.001
	(3.45)	(5.30)	(4.63)		
Pittsburgh Sleep Quality Index (PSQI)	15.60	6.93	7.27	52.20	<.001
	(2.95)	(4.42)	(3.94)		
PSQI Sleep duration	2.33 (1.05)	.73 (1.10)	.60 (.99)	26.48	<.001
PSQI Sleep latency	2.73 (.80)	1.40 (1.24)	1.73 (1.10)	11.97	<.001
Green Paranoid Thoughts Scale: A	46.93	35.20	34.93	11.27	<.001
	(13.27)	(16.89)	(15.60)		
Green Paranoid Thoughts Scale: B	58.27	39.60	38.93	16.11	.001
	(15.93)	(18.80)	(20.80)		
Psychotic Symptom Rating Scale	18.33	15.07	14.00	17.21	<.001
	(2.72)	(3.06)	(4.11)		

Secondary measures					
Cardiff Anomolous Perceptions Scale	18.07	13.20	13.60	5.63	.018
	(10.69)	(9.68)	(9.24)		
DASS: Anxiety Scale	21.80	11.73	13.47	13.13	<.001
	(9.05)	(9.65)	(10.53)		
DASS: Depression Scale	23.13	12.87	16.07	6.35	.008
	(15.41)	(14.37)	(15.88)		

**Table 4:** Pairwise comparisons for pre, post and follow-up ISI and PSQI scores.

Measure		Mean Difference	Std. Error	Sig.	95% CI
ISI Scores	Pre vs. Post	11.80	1.57	<.001	8.43–15.17
	Pre vs. Follow-up	10.73	1.29	<.001	7.97–13.50
	Post vs. Follow-up	1.07	.78	.20	−2.75–.61
PSQI Scores	Pre vs. Post	8.67	1.17	<.001	6.17–11.17
	Pre vs. Follow-up	8.33	1.05	<.001	6.07–10.59
	Post vs. Follow-up	.33	.60	.55	−1.51–.85

**Table 5:** Pairwise comparisons for pre, post and follow-up G-PTS: A; G-PTS: B; PSYRATS scores.

Measure		Mean Difference	Std. Error	Sig.	95% CI
G-PTS: A Scores	Pre vs. Post	11.73	2.65	.001	6.06–17.41
	Pre vs. Follow-up	12.00	3.29	.003	4.95–19.05
	Post vs. Follow-up	.27	2.68	.92	−5.48–6.01
G-PTS: B Scores	Pre vs. Post	18.67	4.28	.001	9.48–27.85
	Pre vs. Follow-up	19.33	4.84	.001	8.95–29.72
	Post vs. Follow-up	.67	1.76	.71	−3.11–4.44
PSYRATS scores	Pre vs. Post	3.27	.60	<.001	1.99–4.55
	Pre vs. Follow-up	4.33	.80	<.001	2.61–6.06
	Post vs. Follow-up	1.07	.88	.25	−.82–2.96

**Table 6:** Pairwise comparisons for pre, post and follow-up CAPS; DASS Anxiety; DASS Depression scores.

Measure		Mean Difference	Std. Error	Sig.	95% CI
CAPS Scores	Pre vs. Post	4.87	1.84	.019	.92–8.81
	Pre vs. Follow-up	4.47	1.85	.03	.49–8.44
	Post vs. Follow-up	.40	.98	.69	−2.5–1.70
DASS: Anxiety Scores	Pre vs. Post	10.07	2.39	.001	4.93–15.20
	Pre vs. Follow-up	8.33	1.91	.001	4.24–12.43
	Post vs. Follow-up	1.73	1.97	.39	−5.95–2.48
DASS: Depression scores	Pre vs. Post	10.27	3.18	.006	3.46–17.08
	Pre vs. Follow-up	7.07	3.29	.05	.02–14.12
	Post vs. Follow-up	3.20	2.28	.18	−8.08–1.68

**Table 7:** Means, standard deviations (in parentheses) and t values for all outcome measures.

	Baseline	Pre-treatment	*t* (df = 6)	*p*-value
Insomnia Severity Index	20.43 (4.47)	21.57 (3.46)	−.738,	.489
Pittsburgh Sleep Quality Index	16.29 (2.36)	17.00 (2.00)	−.645,	.542
Green Paranoid Thoughts Scale: A	52.43 (16.56)	47.71 (13.03)	1.795,	.123
Green Paranoid Thoughts Scale: B	53.00 (17.46)	53.71 (19.28)	−.308,	.768
Psychotic Symptom Rating Scale	19.00 (2.58)	19.29 (2.63)	−1.55,	.172
Cardiff Anomolous Perceptions Scale	15.57 (8.56)	15.71 (9.20)	−.121,	.908
DASS: Anxiety Scale	21.14 (11.89)	21.29 (11.06)	−.040,	.969
DASS: Depression Scale	27.86 (15.13)	28.86 (17.75)	−.512,	.627
